# Thiazides for the prevention of kidney stone recurrence: are they really effective?

**DOI:** 10.1007/s11739-024-03561-3

**Published:** 2024-03-01

**Authors:** Helena Buso, Elena Butera, Daniele Tramontano, Giulia Bandini, The SIMI School of Clinical Research Methodology

**Affiliations:** 1 Department of Medicine-DIMED, University of Padua, Padua, Italy; 2 Department of Internal Medicine, Catholic University of the Sacred Heart of Rome, Rome, Italy; 3 Department of Translational and Precision Medicine, Sapienza University of Rome, Rome, Italy; 4 Department of Experimental and Clinical Medicine, University of Florence, Florence, Italy

## Background

Nephrolithiasis is common worldwide and the incidence and prevalence of kidney stones are increasing globally [1]. The recurrence rate is high and its complications as well as the socio-economic costs are relevant [2]. Kidney stones are heterogeneous in their composition, calcium oxalate and calcium phosphate being the most common. International consensus and guidelines suggest that thiazides should be used in patients with nephrolithiasis for recurrence prevention both in hypercalciuric stone formers and even in normocalciuric patients if non-pharmacological therapies are not effective [3, 4]. However, given the lack of well-designed, randomized, controlled trials, this recommendation is based on heterogeneous and methodologically limited studies. Furthermore, treatment with thiazides can lead to different important side effects and thus the risk/benefit ratio has to be strongly evaluated, especially in otherwise healthy subjects.

## Summary

The NOSTONE trial [5] is a double-blinded, randomized, multicentre, placebo-controlled trial in which patients with recurrent calcium-containing kidney stones were randomly assigned in a 1:1:1:1 ratio to receive hydrochlorothiazide (HCTZ) at a dose of 12.5 mg, 25 mg, or 50 mg once daily or placebo once daily. It was conducted in 12 centres in Switzerland with a duration of treatment of 3 years, except for patients enrolled during the last year of the trial, for whom treatment lasted for 2 to 3 years. The inclusion criteria were age ≥ 18 years, at least two episodes of kidney stones in the past 10 years, and any previous kidney stone that contained at least 50% calcium oxalate, calcium phosphate, or a mixture of both. Patients with secondary causes of kidney stones and those who were treated with drugs that could interfere with the formation of stones were excluded.

The primary endpoint was a composite of symptomatic or radiologic recurrence of kidney stone. At the time of randomization, all patients underwent a low-dose computed tomographic (CT) study limited to the kidney. Symptomatic recurrence, defined as the visible passage of a stone with or without typical symptoms or as the presence of stone that required surgical removal, was assessed at both in-person and telephone follow-up visits. Radiologic recurrence, defined as the appearance of new kidney stones or the enlargement of preexisting one, was assessed at the end of the treatment with a second low-dose CT.

Secondary endpoints were the individual endpoints of symptomatic recurrence and radiologic recurrence or changes in laboratory variables (i.e. total urine volume, urinary sodium, calcium, citrate and oxalate excretion, and urine relative supersaturation ratio of calcium oxalate and calcium phosphate). Safety was also assessed (monitoring of adverse events and clinical laboratory testing).

The sample size was estimated assuming that the risk of symptomatic or radiologic recurrence in the placebo group was 0.20 at 12 months and 0.45 at 36 months.

416 patients were included and followed for a median of 2.9 years. Symptomatic or radiological recurrence occurred in 60 of 102 patients (59%) in the placebo group, in 62 of 105 patients (59%) in the 12.5 mg HCTZ group (rate ratio vs. placebo, 1.33; 95% confidence interval [CI] 0.92 to 1.93), in 61 of 108 patients (56%) in the 25 mg group (rate ratio, 1.24; 95% CI 0.86 to 1.79), and in 49 of 101 patients (49%) in the 50 mg group (rate ratio, 0.92; 95% CI 0.63 to 1.36). There was no relation between the HCTZ dose and the occurrence of a primary endpoint event (p = 0.66).

Of note, patients on HCTZ had lower urinary calcium excretion than those on placebo; however, the urine relative supersaturation ratios for calcium oxalate and calcium phosphate were not consistently lower.

Hypokalemia, gout, new-onset diabetes mellitus, skin allergy, and a plasma creatinine level exceeding 150% of the baseline level were more common among patients on HCTZ than among those on placebo.

According to these data, there was no relation between the hydrochlorothiazide dose and the primary endpoint as the incidence of kidney stones recurrence was similar across the four groups.

The authors conclude that treatment with hydrochlorothiazide did not appear to differ substantially from placebo in preventing the recurrence of kidney stones in patients at high risk for recurrence.

## Strengths of the study

- This study is the first double-blinded, randomized, placebo-controlled trial that evaluated the efficacy and safety of both low dose and high dose of thiazides for the prevention of kidney stone recurrence. This study is well-designed and appropriate for the clinical question. The inclusion and exclusion criteria are deeply defined, all the assumptions for the estimation of sample size are clear and reported in the article, and the primary endpoint is hard and well defined (a composite of symptomatic or radiologic recurrence of kidney stones), which allows an adequate response to the clinical question.
- This study deals with a clinically relevant problem. In fact, there is a high incidence of nephrolithiasis worldwide and, unfortunately, there is a lack of solid evidence on the use of thiazides for this condition.
- The authors made several sensitivity analyses to increase the reliability of the results and exclude possible bias. For example, they made an analysis restricted only to the patients who had no kidney stones at baseline and an analysis in which symptomatic events that had occurred within the first 6 or 12 months after randomization were excluded, and for both of them the recurrence was similar across the four groups.
- The intention-to-treat analysis and the per-protocol analysis showed similar results.

## Weaknesses of the study

- Considering the large number of exclusion criteria, applied to minimize the confounding variables, the sample may not be representative of the target population. This could compromise the transferability of the data into clinical practice.
- There was an underrepresentation of women, only 20% of the sample, and of black and Asian patients which were around 1% of the sample.
- There was a high incidence of no adherence: 26% in the placebo group, 15% in the 12.5 mg HCTZ group, 24% in the 25 mg HCTZ group, and 26% in the 50 mg HCTZ group. These factors may cause an underestimation of treatment efficacy.

## Question marks

- The maximum dosage of HCTZ used in this study was 50 mg once daily; however a dosage of 100 mg and a twice daily dose regimes are also used for the prevention of recurrence kidney stones. Thus, it cannot be ruled out that a higher dosage (e.g. 100 mg), or a twice daily dose administrations or long-acting formulations might have provided different results. However, higher doses may have significant side effects.
- We wonder if there might be a possible beneficial effect of hydrochlorothiazide in specific subgroups of patients (i.e. patients with a higher disease severity and patients with hypercalciuria). Future ad hoc studies are needed.

## Conflict of interest

The authors received no financial support for the research, authorship, and/or publication of this article. This study was supported by the Swiss National Science Foundation (grant 33IC30_166785) and Inselspital, Bern University Hospital, University of Bern.

## Clinical bottom line

In our opinion this study does not support the use in clinical practice of thiazides for the prevention of kidney stone recurrence, given the non-significant difference in the efficacy vs placebo,especially thinking about potential side effects. However, given the limitations previously mentioned, further studies could be useful to better clarify some important aspects of this topic and identify possible groups of patients who may benefit from HCTZ.

## Data Availability

This is a cutting edge. No dataset was generated for the publication of this article.
